# Fuzzy Modelling for Human Dynamics Based on Online Social Networks

**DOI:** 10.3390/s17091949

**Published:** 2017-08-24

**Authors:** Jesus Cuenca-Jara, Fernando Terroso-Saenz, Mercedes Valdes-Vela, Antonio F. Skarmeta

**Affiliations:** Department of Communications and Information Engineering, University of Murcia, Murcia 30100, Spain; jesus.cuenca1@um.es (J.C.-J.); mdvaldes@um.es (M.V.-V.); skarmeta@um.es (A.F.S.)

**Keywords:** fuzzy clustering, urban mobility, online social networks

## Abstract

Human mobility mining has attracted a lot of attention in the research community due to its multiple implications in the provisioning of innovative services for large metropolises. In this scope, Online Social Networks (OSN) have arisen as a promising source of location data to come up with new mobility models. However, the human nature of this data makes it rather noisy and inaccurate. In order to deal with such limitations, the present work introduces a framework for human mobility mining based on fuzzy logic. Firstly, a fuzzy clustering algorithm extracts the most active OSN areas at different time periods. Next, such clusters are the building blocks to compose mobility patterns. Furthermore, a location prediction service based on a fuzzy rule classifier has been developed on top of the framework. Finally, both the framework and the predictor has been tested with a Twitter and Flickr dataset in two large cities.

## 1. Introduction

One of the most important social phenomena of the last decades has been the endless transference of population from rural areas to urban ones. As a matter of fact, it is foreseen that 9% of the worldwide population will reside in 41 megacities in the short term [1]. As a result, metropolises are now much more complex and dynamic than ever before. This never-ending growth imposes new challenges on administrators and planners in order to provide city dwellers with an acceptable welfare state, such as air quality control, intelligent public transportation services or efficient allocation of energy resources.

When it comes to tackling all these challenges, a comprehensive understanding of the human dynamics within cities plays a paramount role [2]. In this frame, several works have already put forward the impact of human mobility on understanding or even predicting the economic development and social conditions of a city [3,4]. For that reason, the study of mobility patterns that define such dynamics have been addressed in many different forms. They can be split into three different trends depending on the source of data under consideration, namely, (1) travel survey, (2) wireless sensor mechanisms [5] and (3) mobile-phone network [6] methods. Despite the fact that these sources of mobility data have given rise to suitable and useful results, all of them suffer from serious drawbacks in terms of costs associated with their gathering and availability because of privacy, security or proprietary reasons.

Due to these drawbacks, a novel trend in the mobility research domain has started to consider Online Social Networks (OSNs) as a suitable source of data [7]. OSNs now constitute virtual worlds where users share their activities or interest with their online friendships. These worlds intersect with the real one by means of locations that act as connectors. This is mainly because most mainstream OSN platforms, such as Twitter [8], Facebook [9] or Flickr [10], now include location-based capabilities into their web or smartphone’s applications that have enabled the geo-tagging of most of their documents. Hence, when a user submits, for example, a tweet on Twitter or a post on Facebook, the textual content that he or she personally writes is automatically enriched with the spatial coordinates of his current location.

This way, the combination of the popularity of these platforms (the number of active users of social media reached 2.031 billion in 2015 [11] along with the widespread existence of personal handheld devices, generates an unprecedented wealth of location data. What is more important, unlike previous sources, is that this data is highly accessible by using the Application Programming Interfaces (APIs) provided by their own OSN platforms. However, it is also true that OSN data tends to be more sparse than traditional mobility feeds due to the slow pace that most users exhibit when it comes to posting geo-tagged documents [12]. This makes it quite difficult to compose high-resolution mobility logs from such data. In addition to that, only a low percentage of OSN documents is actually geo-tagged. As a matter of fact, only 1.6 percent of Twitter users actually have the automatic geo-tagging functionality turned on [13]

An important line of work within the OSN-based mobility mining intends to uncover the usage of different social areas of a city by applying several clustering algorithms to geo-tagged OSN data. In these works, each identified cluster is regarded as a different area of interest of the target city [14,15,16]. Nevertheless, we have observed that existing solutions in this domain do not generally take into account all the characteristics that OSNs have in terms of mobility. This is substantiated in the following common limitations which present solutions that usually incur.

- First of all, most OSN-based clustering algorithms frequently only use the spatial meta-data of documents to generate the clusters. However, the textual content of the documents, what users have actually created by themselves, is not considered for the clusters’ generation. As a result, current solutions do not actually take full advantage of the underlying knowledge contained in OSN data sources.
- Secondly, the user-generated nature of OSN data makes it inherently noisy and imprecise. For example, Flickr photographs are usually geo-tagged with the place where they were taken that might not be exactly the same place where the true landmark is located. Existing solutions generally do not take into account such inner characteristics during the clustering process. It will have an impact on the generation of mobility patterns though.
- Finally, current mechanisms focus on extracting general mobility information related to a particular urban area without distinguishing the time of the day in which the information was generated. Hence, they do not study the relationship between the moment of the day at which social-media documents are posted and its associated spatial place. This missing information could provide a global vision of the movement of a population along a day. Therefore, these works are not taking full advance of social-media datasets.

In this context, the present work introduces a novel mechanism for human mobility characterization that exploits all the benefits that OSN data can bring in terms of its spatial, temporal and textual aspects. In order to enable this full characterization, the proposal follows a fuzzy-modelling approach that considers the inherent uncertainty associated with OSN data in a formal manner. This is instantiated in an OSN-based mobility framework that provides a complete solution to the limitations listed before. In that sense, the usage of the spatial and textual content of OSN data makes the resulting model provide not only the location of the social areas of the city but also a set of labels associated with each cluster describing its predominant activity or landmark giving rise to the most valuable information.

Finally, in order to study the feasibility of the proposal, a lightweight location predictor has been developed on top of the proposed framework. This service profits from people displacement between clusters in different time slots so as to forecast the location where an OSN user is going to submit his next document. These types of location predictors are instrumental for many mobility operators [17]. In order to be consistent with the mobility framework, this predictor also follows a fuzzy-rule approach in order to infer the predicted outcome. Furthermore, it has been designed by considering the widespread nature of OSN data mentioned before, as it does not rely on long mobility records to make a prediction. Both the framework and the predictor have been evaluated with a large dataset containing documents from Twitter and Flickr platforms.

The paper is structured as follows: Section 2 provides a brief overview of the proposal. Then, Section 3 looks into the framework, including its architecture and functional modules. Section 4 describes the predictor service built on top of the framework. Section 5 provides an evaluation of some of the features of the platform. Section 6 provides a comparative of our work with the existing state of the art; and Section 7 concludes the paper with some final remarks and conclusions.

## 2. System Overview

**Figure 1:**
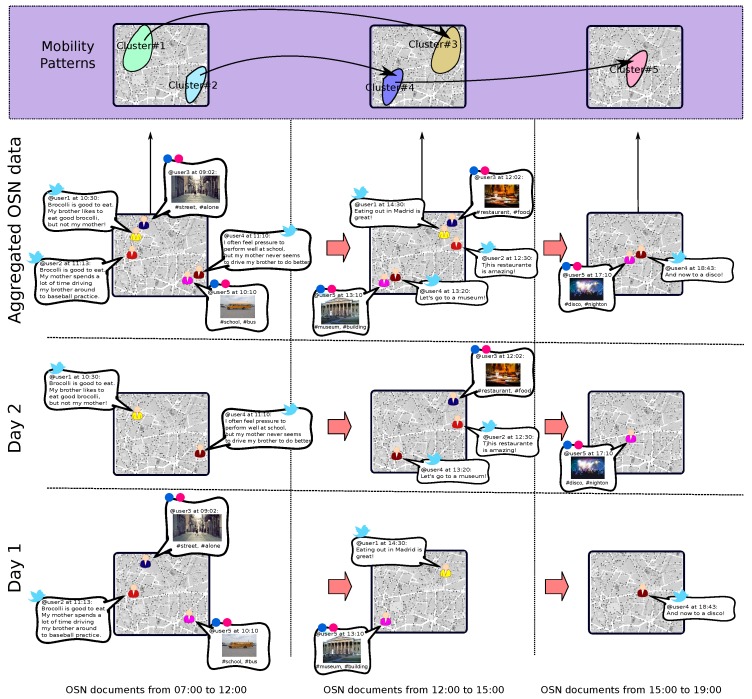
Approach overview.

Figure 1 depicts a general overview of the proposal. From the raw OSN documents published at different time periods and days depicted at the bottom of the figure, the present solution’s outcome is shown on top of it. As we can see, the proposal is able to identify the spatial areas of a city with a high level of OSN activity at different time periods (e.g., clusters 1 and 2 during the morning or cluster 5 during the afternoon). For this task, we have integrated the Gustafson–Kessel (GK) clustering algorithm [18] and the Hierarchical Dirichlet Process (HDP) [19].

Furthermore, this fuzzy-clustering process is not monolithic, but it is launched for different time slots. In order to keep on with the fuzzy approach, these slots are defined by fuzzy sets. This temporal aspect of the solution makes it possible to detect how the active social areas move across the city’s throughout time.

On the basis of these clusters, the human movement between time periods can be established. For instance, according to the figure, most of the people at cluster 1 in the morning moves to cluster 3 in the afternoon.

## 3. The Fuzzy Modelling Process

In this section, the fuzzy modelling solution to extract the mobility patterns of a city is put forward. In brief, the proposed solution follows a four-step processing pipeline:

- Firstly, collect and filter the OSN documents from the target OSN platforms.
- Secondly, transform the clean documents into a format able to define a similarity distance between OSN documents integrating both their spatio-temporal and their textual features.
- Next, perform the fuzzy clustering over the product space of input features generated on the basis of the transformed documents to discover regions with high human activity.
- Finally, compute the movement of people between the discovered clusters defining the mobility patterns of the area under study.

**Figure 2:**
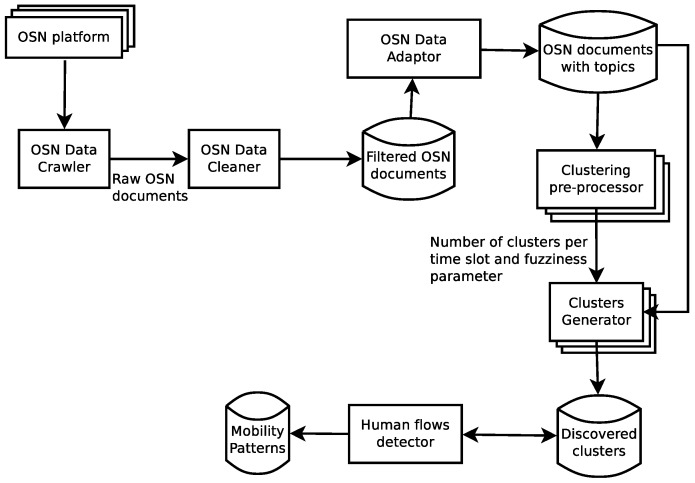
Architecture of the solution.

Figure 2 shows different modules that realize the aforementioned process, each one representing a different step in the analysis of OSN documents. The following sections state each of these steps in detail.

For the sake of clarity, Table 1 summarizes the key acronyms used in the following sections.

### 3.1. OSN Data Collection and Cleaning

The first step in the processing loop is to gather the needed documents from the target OSN platforms for their further analysis related to the urban area under study A. Many of these platforms already provide open Application Programming Interfaces (APIs) that can be used in order to gather their publicly visible documents. Depending on the platform under consideration, these documents will take the form of tweets in the case of Twitter, posts on Facebook or labelled photographs on Flickr.

Despite this variety, the present work relies on a uniform view of the gathered documents. Hence, a raw OSN document is a tuple d=<u,p,l,t,c>, where *u* is the OSN user who actually posted the document, *p* the host OSN platform, *l* the spatial spatial coordinates {x,y} at which *d* was posted, *t* the timestamp of the submission and *c* the textual content of the document directly written by *u*.

This way, the OSN data crawler (see Figure 2)) focuses on keeping only geo-tagged OSN documents, discarding the ones that do not include a location *l* among their meta-data. Moreover, the current work only considers the textual content of a document discarding other types like images, sounds or videos.

Once the extraction of the OSN documents has been completed, it is necessary to clean the collected dataset so that it eventually contains accurate human mobility information. In that sense, OSNs usually comprise a significant proportion of redundant and useless (spam) content that might disturb the obtained results. For example, it is reported that about 10% of Twitter content is spam [20].

For this reason, documents from OSN accounts representing companies, institutions and so forth or having an unusually high posting frequency are removed from the collected dataset. In addition to that, consecutive documents posted by the same user *u* close in time and space are merged into a single document. Next, the textual content *c* of each document *d* is cleaned by removing its stop words and performing word stemming.

**Figure 3:**
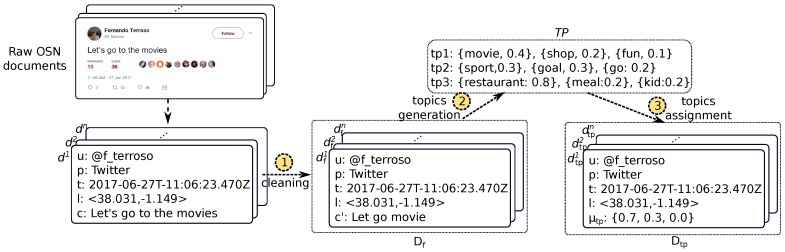
Example of pre-processing of the Online Social Network (OSN) document.

As Figure 3 depicts, this initial stage results in a database $D_{f}=\left\{d_{f}^{1},d_{f}^{2},..,d_{f}^{n}\right\}$ of filtered OSN documents $d_{f}=<u,p,l,t,c^{\prime }>,$ where $c^{\prime }$ means the textual content of the document without stop words and the rest of words in their root form. This repository solely contains documents from actual citizens, and each document represents a meaningful displacement of such citizens either in the space or the time dimension.

### 3.2. OSN Data Transformation

Once the OSN documents have been collected and cleaned, the next step is to transform such documents to make them compatible with a distance metric that allows for measuring the similarity between documents. The definition of this measurement is paramount for our approach to properly process OSN data by means of a clustering algorithm. In that sense, the OSN Data Adaptor module (see Figure 2) transforms the textual content $c^{\prime }$ from the filtered documents in $D_{f}$ into a vector-based format.

For this goal, we have made use of the Hierarchical Dirichlet Process (HDP) [19]. HDP is a non-parametric Bayesian mechanism that has been widely used in the information retrieval field in order to uncover the latent topics of sets of documents. Unlike the well-known Latent Dirichlet Allocation (LDA) model [21], HDP does not need to know in advance the number of topics to be generated. On that contrary, it is able to automatically learn the number of topics to be detected over the document corpus.

This way, a HDP instance is fed with a corpus $D_{f}\left(c^{\prime }\right)$ comprising the textual content $c^{\prime }$ of all the documents in $D_{f}$. This instance returns a distribution of *m* topics TP of such a corpus defined as follows (see arrow 2 in Figure 3),

$$\mathrm{TP}=\{tp_{1},tp_{2},..,tp_{m}\;|\;tp_{i}=\left\{\left\{p_{i}^{1},w_{i}^{1}\right\},\left\{p_{i}^{2},w_{i}^{2}\right\},..,\left\{p_{i}^{k},w_{i}^{k}\right\}\right\}\;\forall i\;\in \left[1,m\right]\}.$$

As we can see, each generated topic tp is represented as a probability distribution $\left\{p^{1},..,p^{k}\right\}$ over a word subset $\left\{w^{1},..,w^{k}\right\}$ in $D_{f}\left(c^{\prime }\right)$.

Once the topics have been uncovered, the HDP model also allows to know the membership of a particular document to each of these topics. We leverage this feature so as to re-format the textual content of the documents in $D_{f}$. This way, each document $d_{f}$ is replaced with a new document with topics $d_{tp}=<u,p,l,t,\mu_{tp}>,$ where $\mu_{tp}=\left\{\mu_{tp}^{1},\mu_{tp}^{2},..,\mu_{tp}^{m}\right\},$ and where $\mu_{tp}^{i}$ represents the membership of the document to the i-th topic and $\sum_{i=1}^{m}\mu_{tp}^{i}=1$ (see arrow 3 in Figure 3).

At this point, the original textual content of an OSN document *c* has been transformed to a numeric vector $\mu_{tp}$ over which we can easily define a distance metric. Finally, the resulting dataset $D_{tp}=\left\{d_{tp}^{1},d_{tp}^{2},...,d_{tp}^{n}\right\}$ is stored in a repository as Figure 2 depicts.

### 3.3. Fuzzy Cluster Generation

The next step in the mobility patterns discovery focuses on executing the fuzzy clustering algorithm to detect the areas of social activity of a city. In more detail, we have applied the Gustafson–Kessel (GK) clustering algorithm [18]. GK is one of the most commonly used solutions to extract fuzzy clusters from a set of data. Unlike other well-known algorithms like Fuzzy C-Means [22], GK is able to detect elliptical clusters instead of spherical ones. Therefore, if the data is distributed in different clusters, and they are of different shapes and orientations, the GK is more likely to discover the real underlying structure of data than using an algorithm that imposes, for example, spherical shapes that could not be present in the data. This is quite convenient in the present domain due to the fact that OSN documents do not usually follow a homogeneous distribution in urban environments [23]. How this algorithm has been adopted in this work is stated in the following sub-sections.

#### 3.3.1. Input Selection

This work relies on the assumption that active areas of a city are not the same during the whole day, but they change through time. This has been already pointed out by existing literature in human pattern mining [24,25]. For example, business parks or university campus attract a lot of activity during the morning and afternoon, whereas residential areas or shopping malls have a high level of human activity at later hours.

**Figure 4:**
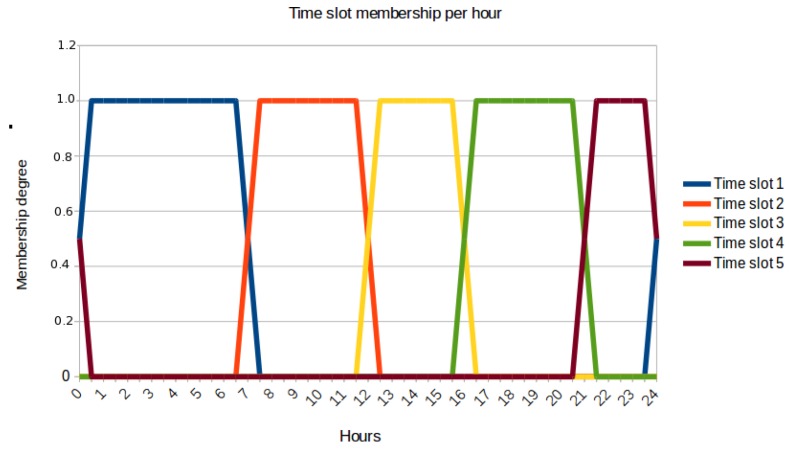
Fuzzy sets defining the time periods of a day.

For that reason, we manually split the 24-hour period of a day into five different time slots. Such a time division is consistent with previous ones proposed in the mobility mining field [26,27,28]. In order to be compliant with the fuzzy modelling approach of the solution, these time slots were defined as trapezoidal fuzzy sets as Figure 4 depicts. As a result, each document $d_{tp}\in D_{tp}$ will have a particular membership degree to each of these slots depending on its timestamp field *t*.

Then, we launch a different GK instance for each slot. Hence, the generated clusters in each of these individual processes will uncover the social areas related to its target time period. For example, the GK instance for time slot 2 will detect the active social areas for the time period between 6:00 a.m. and 1:00 p.m. approximately according to Figure 4, whereas the GK instance for time slot 3 will cover from 11:00 a.m. to 5:00 p.m. This composes a fuzzy time period between 11:00 a.m. and 1:00 p.m. that is properly handled by the current approach.

Concerning the product space of input features of each GK instance, in our case, this will be L×M(TP), where L is the space comprising the location coordinates of OSN documents and M(TP) the membership degree of OSN documents to the uncovered topics. Other fields of an OSN document like the user *u* or the host OSN platform *p* are not considered by the clustering algorithm.

#### 3.3.2. Algorithm Adaptation

Given the dataset $D_{tp}\left(l,\mu_{tp}\right)\subset L\times M\left(\mathrm{TP}\right)$, the pseudo-code of the GK algorithm to generate the clusters for a time slot *s* is described in Algorithm 1.

From such a code snippet, we can see that the general structure of the algorithm is very similar to its standard version. However, we have incorporated a few but meaningful modifications to adapt the algorithm to the particularities of our work.

Firstly, the computation of the clusters’ prototypes and covariance matrices (Equations (2) and (3)) considers not only the membership of the *k*-th document $d_{tp}^{k}$ to the *i*-th cluster, but also the membership degree of such a document to the target time slot *s*, $\delta_{k}^{s}$. This way, the contribution of each OSN document to the clusters generated for a time slot is proportional to its closeness in time.

Secondly, the computation of the distance between a document $d_{tp}$ and a cluster prototype $v_{i}^{\left(j\right)}$ ($d_{tp}^{k}-v_{i}^{\left(j\right)}$) (Equations (3) and (4)) needs to consider both the spatial and textual aspects of the documents. For this reason, such distance is calculated as a combination of two different metrics:

(1) $$\begin{array}{cc} d_{tp}-v_{i}= & \alpha_{loc}\times \frac{dist_{harv}\left(d_{tp}\left(l\right),\;v_{i}\left(l\right)\right)}{max_{dist}}\;+\\ & \left(1-\alpha_{loc}\right)\times sim_{coisine}(d_{tp}\left(\mu_{tp}\right),\;v_{i}\left(\mu_{tp})\right). \end{array}$$

**Algorithm 1:** Gustafson–Kessel Algorithm
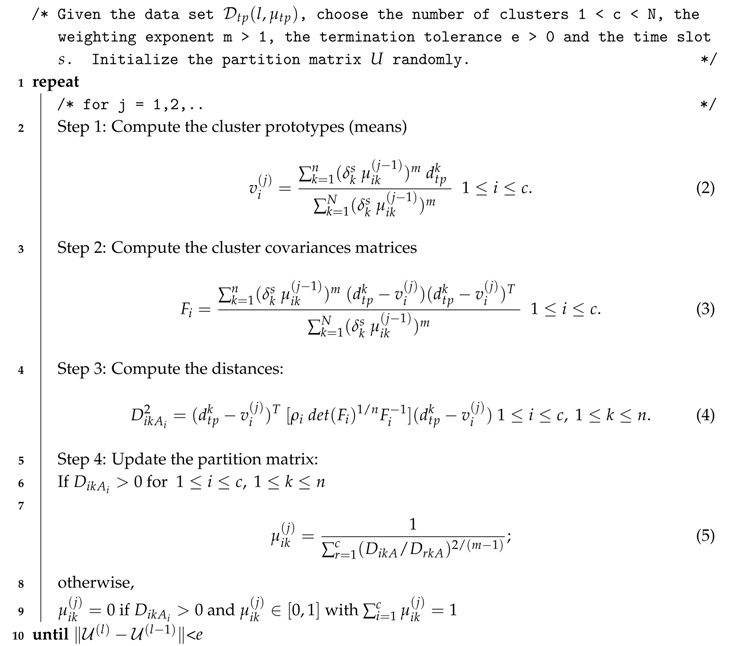


As we can see, the distance between a document and a centroid is composed of two aspects. For the location feature, we use the haversine formula [29] that determines the spherical distance between the coordinates in *l*. As for the textual features, we compute the coisine similarity [30] between the vectors comprising the membership of each element to the topics previously generated by the HDP model (see Section 3.2).

Moreover, the weighting parameter $\alpha_{loc}$ (∈[0,1]) allows for controlling the actual contribution of both features to the similarity computation. This way, we are able to generate just spatio-temporal patterns ($\alpha_{loc}=1$) or just patterns reporting semantic features ($\alpha_{loc}=0$). In that sense, the haversine distance is normalized with respect to a maximum distance between two locations in A so that both the spatial and textual features can contribute equally to the similarity computation.

All in all, by means of the membership degrees for the time slots $\delta_{k}^{s}$ and the multi-variate distance formula of Equation (1), we are able to smoothly integrate the temporal, spatial and textual features of the OSN documents in the clustering process.

Finally, since the cluster prototypes from the aforementioned process are generated on the basis of the *l* and $\mu_{tp}$ fields, they represent the location center of areas with a high level of human activity and the relevant topics from TP associated with such areas. In that sense, five sets of these clusters are composed $C_{ts}=\left\{C_{1},C_{2},C_{3},C_{4},C_{5}\right\}$, one for each time slot.

#### 3.3.3. Initial Number of Centroids and Weighting Exponent Specification

One of the most important limitations of most clustering algorithms is that is not easy to determine its parameters, the number of clusters to be generated (*c*) and the weighting exponent (*m*). This last parameter determines the fuzziness of the clusters. The larger the value of *m* is, the more overlapped the clusters are. In the current work, instead of establishing both parameters a priori, a suitable value of *m* and *c* is automatically obtained from the data as it is done in [31].

The mechanism is based on a cluster validity measure that takes into account the compactness of and the separation between clusters (see Appendix A). Basically, for every number of clusters *c*, *m* is incremented in $m_{inc}$ until the cluster validity criterion is fulfilled (see Appendix B).

### 3.4. Human Mobility Detection

The clusters generated in the previous step are the basis to finally compose the flows that define the human movement of the area of interest. Basically, this composition is done by following a bottom-up approach, we firstly categorize the mobility of each unique user *u* and then aggregate such individual data to compose a crowd-based information representing the whole mobility of the target area. This process is summed up in Algorithm 2.

To start with, the algorithm detects the most representative cluster for each user in each of the five pre-defined timeslots (lines 2–10). This is done by obtaining the cluster in each set $C_{i}$ where the user’s documents have the highest membership degree on average. For this computation, we need the set of partition matrices per time slot $U_{ts}=\left\{U_{1},U_{2},U_{3},U_{4},U_{5}\right\}$ generated by the GK instances comprising the membership of the documents in $D_{tp}$ to each of the clusters.

At the end of this process, $p_{user}$ contains the mobility pattern of the target user in terms of his movement in between time slots. It should be noted that this approach aggregates the different documents published by the user during the entire period under study (see Figure 1). This is particularly useful in the OSN domain where data scarcity makes it rather challenging to compose users’ paths covering different time slots in a single day.

Table 2 shows some examples of this variable. This way, we can see that user1 usually stays close to cluster $A_{1}$ during time slot 1 and moves to cluster $B_{2}$ at time slot 2. Similarly, user5 moves from cluster $B_{2}$ to $C_{3}$ at time slot 3. It might happen that there is no information for a user given a particular time slot (like user3 for time slot 1). This is because a user does not publish documents during that time slot and, thus, the algorithm is not able to extract any representative cluster.

Given such individual patterns, the second part of the algorithm focuses on composing the aggregated patterns describing the movement of the whole urban area under study (lines 11–63). To do so, we firstly compute the number of occurrences of each cluster and the number of transitions between clusters at different time slots (not necessarily consecutive) in the individual patterns (lines 11–20). Then, we normalize transitions’ counting with the number of occurrences of the origin cluster (lines 21–26). As a result, we obtain the rates of users that move from one cluster to another at a different time slot. Such rates are represented as a multi-dimensional table $P_{A}$ in Algorithm 2. This way, $P_{A}^{\left(2,3\right)}$ comprises the transition rates from clusters in time slot 2 to clusters in time slot 3. 

**Algorithm 2:** Pattern discovery algorithm
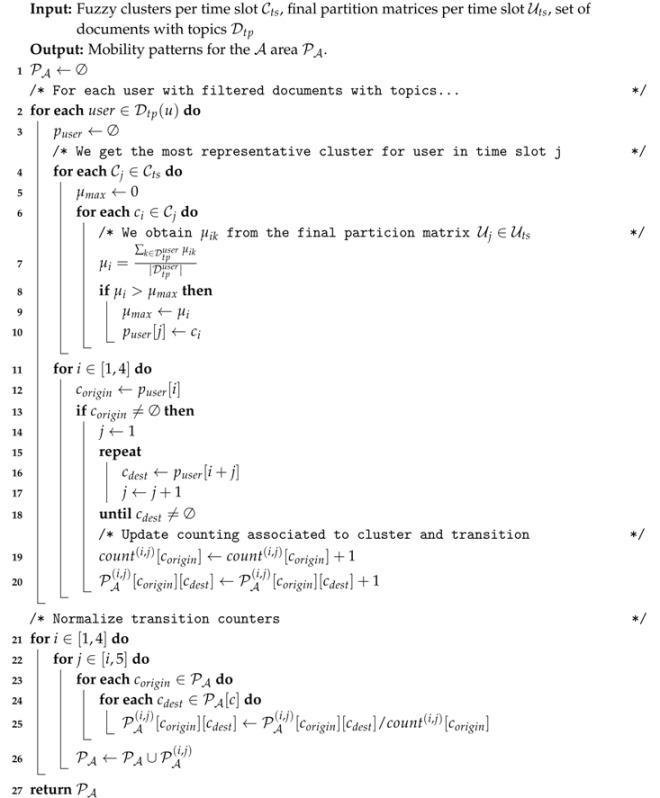


Going back to our illustrative example of Table 2, if we stick to time slots 1 and 2 (night and morning periods according to Figure 4), the aforementioned process will detect that 66% of users who spend the night near cluster $A_{1}$ then move to $B_{2}$ during the morning ($\frac{P_{A}^{\left(1,2\right)}\left[A_{1}\right]\left[B_{2}\right]}{count[A_{1}]}=\frac{2}{3}=0.66$).

Although this approach focuses on extracting patterns between consecutive time slots, we can easily use them to compose longer patterns by just linking the rows and columns of table $P_{A}$. Such multi-timeslot patterns take the form of a sequence $\langle P_{A}^{\left(1,2\right)}\left[X_{1}\right]\left[X_{2}\right]\to P_{A}^{\left(2,3\right)}\left[X_{2}\right]\left[X_{3}\right]\to ...\to P_{A}^{\left(4,5\right)}\left[X_{4}\right]\left[X_{5}\right]\rangle ,$ where $\langle X_{i},X_{i+1}\rangle$ are clusters in consecutive timeslots with a certain percentage of movement. This approach is different than well-known solutions for trajectory pattern extraction [32] based on the Frequent Sequential Pattern (FSP) problem [33]. This type of algorithm is designed to operate with high-resolution spatio-temporal trajectories, where the target moving object frequently reports their current location. As a result, each individual trajectory may comprise hundreds or thousands of different locations. On the contrary, OSN data tends to provide more spread and coarse-grained routes that might not be dense enough to extract accurate patterns. Furthermore, FSP-based solutions provide information about the overall frequency (support) of the extracted pattern. However, our solution allows for knowing the particular percentage of users moving between each pair of clusters providing more detailed mobility information.

All in all, we follow a memory-based approach to represent the mobility patterns by table $P_{A}$. In that sense, the dimensions of such a table corresponds to the total number of clusters generated by the clustering process. Such a number is calculated by the data-driven approach described in Section 3.3.3. As a side effect of this process, we optimize $P_{A}$ dimensions avoiding their underestimation or overestimation, which will, in turn, affect the optimal representation of the global patterns due to data-scarcity issues and the cost-effective allocation of resources for its storage.

Finally, bearing in mind the known limitations of existing OSN-based mobility mining solutions pointed out in Section 1, our approach proposes several mechanisms to deal with them:

- Firstly, regarding the underestimation of the textual content of OSN data, such a content is smoothly fused in the clustering process as topic-based features of the OSN documents as described in Section 3.2.
- Secondly, as far as the noisy nature of human-generated data is concerned, the combination of fuzzy clustering and HDP avoids defining hard boundaries indicating whether a document belongs or not to a certain cluster (see Section 3.3.2). On the contrary, the adopted approach defines fuzzy boundaries that makes it suitable for OSN documents where either its textual or location content is noisy or imprecise, which makes it difficult to assign it to only one cluster.
- Lastly, as for the monolithic mobility patterns in terms of time evolution, the split of the mobility mining in different time slots within a day allows for extracting more time-aware, and thus more dynamic, mobility patterns.

## 4. Location-Based Predictor Service

In order to test the feasibility of our approach, we have developed a prediction service able to estimate where an OSN user is going to post his or her next document making use of the learned mobility patterns.

Since these patterns have been defined on the basis of a palette of fuzzy clusters, the present prediction service leverages such fuzzy approach and its design takes the form of a fuzzy IF-THEN classifier. Fuzzy classifiers have been successfully applied to pattern classification tasks [34,35]. Concretely, the model for the current problem has the form:

$$\mathrm{IF}d_{tp}\mathrm{is}c_{origin}^{1}\mathrm{THEN}c_{dest}^{1}=\frac{\sum_{k}P_{A}\left[c_{origin}^{1}\right]\left[c_{k}\right]\times c_{k}}{\left|k\right|},\;\mathrm{IF}d_{tp}\mathrm{is}c_{origin}^{2}\mathrm{THEN}c_{dest}^{2}=\frac{\sum_{k}P_{A}\left[c_{origin}^{2}\right]\left[c_{k}\right]\times c_{k}}{\left|k\right|},\;\ldots \;\mathrm{IF}d_{tp}\mathrm{is}c_{origin}^{r}\mathrm{THEN}c_{dest}^{r}=\frac{\sum_{k}P_{A}\left[c_{origin}^{r}\right]\left[c_{k}\right]\times c_{k}}{\left|k\right|},$$

where $c_{origin}^{i}$ is the fuzzy set for the *i*-th rule, $c_{dest}^{i}$ is the partial output of the *i*-th rule, and being i=1,…,r, *r* being the number of rules.

As regards the fuzzy reasoning mechanism, it is such that the firing strength $\tau_{i}$ for the *i*-th rule given an input OSN document $d_{tp}$ is obtained by the expression:

$$\tau_{i}\left(d_{tp}\right)=\mu_{i}\left(d_{tp}\right),$$

where $\mu_{i}$ denotes the membership degree to the associated fuzzy cluster. Then, the partial output $c_{dest}^{i}\left(d_{tp}\right)$ is given by:

$$c_{dest}^{i}\left(d_{tp}\right)=\tau_{i}\left(d_{tp}\right)\times c_{dest}^{i},$$

and the partial outputs are combined to generate the final prediction $c_{dest}$ of the system according to:

$$c_{dest}=\frac{\sum_{i=1}^{r}\tau_{i}\left(d_{tp}\right)\times c_{dest}^{i}\left(d_{tp}\right)}{\sum_{i=1}^{r}\tau_{i}\left(d_{tp}\right)}.$$

**Figure 5:**
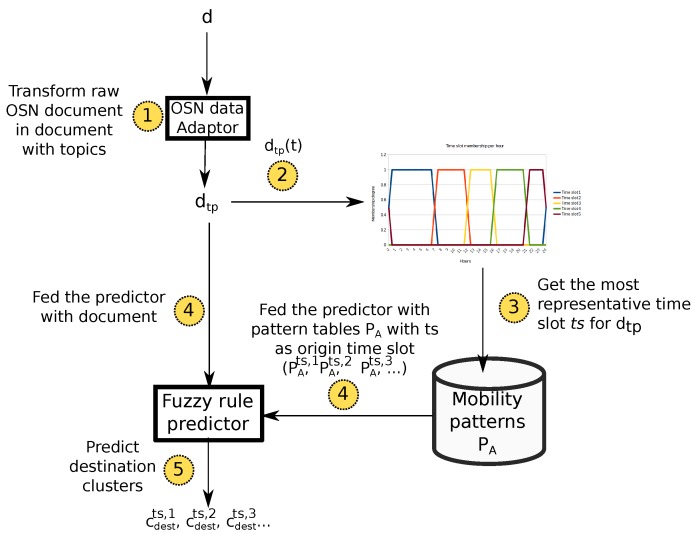
Workflow of the prediction mechanism.

For the sake of clarity, Figure 5 summarizes the whole prediction loop. This way, this system takes the last raw OSN document *d* published by a user and transforms it to a document with topic $d_{tp}$ (see Figure 3) (arrow 1 in Figure 5). Next, the mechanism infers the most representative time slot ts that such a document belongs to (arrows 2 and 3). This allows for selecting the sub-tables of $P_{A}$ with such a time slot as origin ($P_{A}^{\left(ts,x\right)}$).

The fuzzy classifier is fed at different times with the selected sub-tables in order to generate predictions for different time slots (arrows 4). This way, the system is able to provide the potential destination of the target user for several time horizons. Due to the spatial and topic-related information contained in each cluster, predicted clusters will not only indicate the potential future location of the user but also semantic information about such a location.

Finally, as we can see, the predictor takes under consideration the data-sparsity problem of OSN platforms. In that sense, several works already state that most users tend to post roughly one or two OSN documents per day [12]. As we have seen, the present solution does not rely on a long sequence of previously-visited clusters by a user in order to infer a potential destination. On the contrary, it only makes use of the most recently-visited cluster. Hence, the rationale of this approach is to provide a solution suitable for a wide range of users.

## 5. Evaluation of the Proposal

In this section, we state the main findings from the evaluation of the framework along with the prediction service.

### 5.1. Experiment Setup

#### 5.1.1. Implementation Details

Most of the components of the framework and the predictor have been implemented ad hoc for the present project using the Python and C++ programming languages. However, for the cleaning of the textual content of the documents, we have made use of the Natural Language Toolkit (NLTK) [36] and the gensim library [37] for the implementation of the HDP model.

#### 5.1.2. Datasets

To evaluate our proposal, we used three different OSN datasets targeting two large metropolises: Madrid (Spain) and New York (United States). Such datasets comprised documents from two different platforms: Twitter and Flickr. Whilst the Twitter data was obtained by using the Twitter Crawling API [38], the Flickr documents are part of the Yahoo Flickr Creative Commons 100M public dataset [39].

This way, we study the performance of our proposal by using documents from two different platforms in terms of usage at different cities. While Twitter is characterized as a way to communicate with friends, follow people of interest and share your views along with a minute news provider, Flickr is basically a social platform for photograph sharing. In more detail, we just keep for each city the geo-tagged documents from these two platforms that fit into the spatial polygon defined for each city in OpenStreetMap [40]. Furthermore, the three datasets cover a three-month time period. Table 3 summarizes the details of these three datasets.

**Figure 6:**
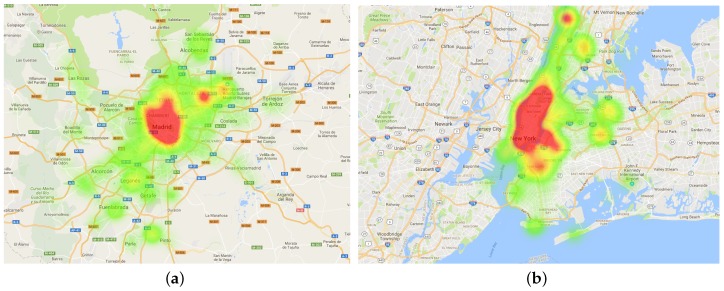
Heat map of documents density of the evaluated datasets. (**a**) Madrid (MD) Dataset (Twitter + Flickr); (**b**) New York (NY) dataset (Flickr).

Finally, Figure 6 shows the heat map of the datasets showing the spatial density of OSN documents in the two target cities. From such maps, we observe the direct correlation between the density of documents and their distance with respect to the center of the cities. This is because the city centers usually contain a high population density or an intense social activity.

### 5.2. Dataset Cleaning

The first step in our evaluation was to perform the dataset cleaning as stated in Section 3.1. In that sense, the cleaning mechanism discarded any new document from a user published in less than 60 min or 1 km distance from his previous document. As we can see from Table 4, there is a meaningful difference in the cleaning results depending on the city. In particular, the rate of irrelevant documents was much higher in the Madrid (MD) dataset than in New York (NY). In our view, this is because the purpose and mechanics of Twitter favour the emergence of spam users that flood the network with irrelevant content. In that sense, only 30 different users generated about 30% of the content in the original MD dataset. On the contrary, the NY dataset was less affected by this spam flooding issue.

Furthermore, Table 5 also shows the distribution of documents per time slot. In that sense, for this experiment, we have used the same time-slot fuzzy sets of Figure 4. This computation has been done by just simply assigning a document to its most representative time slot (the one with the highest membership degree).

As we can see, timeslots 3 and 4 representing the afternoon and evening periods contain the majority of the documents. However, dataset NY comprising only Flickr data have a more balanced distribution of documents than the MD dataset combining Twitter and Flickr. These differences might be due to multiple reasons. Firstly, social habits in the each of the cities area quite different. In that sense, it is reported that Spaniards used to go to bed later than any other European country [41]. This explains that the percentage of documents during late evening is much higher in MD than in the other two cities. Furthermore, most Flickr users are actually tourists visiting a different city [42]. This could explain the more homogeneous distribution of documents across time slots and the higher activity during the morning period (when the majority of landmarks of the city are open).

### 5.3. Cluster Generation

Once the data cleaning was performed, we launched the clustering process to the resulting datasets in order to uncover the active social areas of the cities. For their generation, we set the $\alpha_{loc}$ parameter to 0.65 so as to have a balanced trade-off between the location and semantic information from the documents (see Section 3.3.2).

Table 6 shows the number of clusters automatically detected by the data-driven mechanism described in Section 3.3.3. In that sense, this number of clusters was the same for all the time slots.

As we can see, the number of total documents seems not relevant for the number of clusters generated as the NY dataset gave rise to 23 clusters, whereas the MD dataset, which has a larger number of documents, was structured in 20 clusters. However, a correlation does exist between the spatial region covered by each dataset (set Table 3) and the number of final clusters.

**Figure 7:**
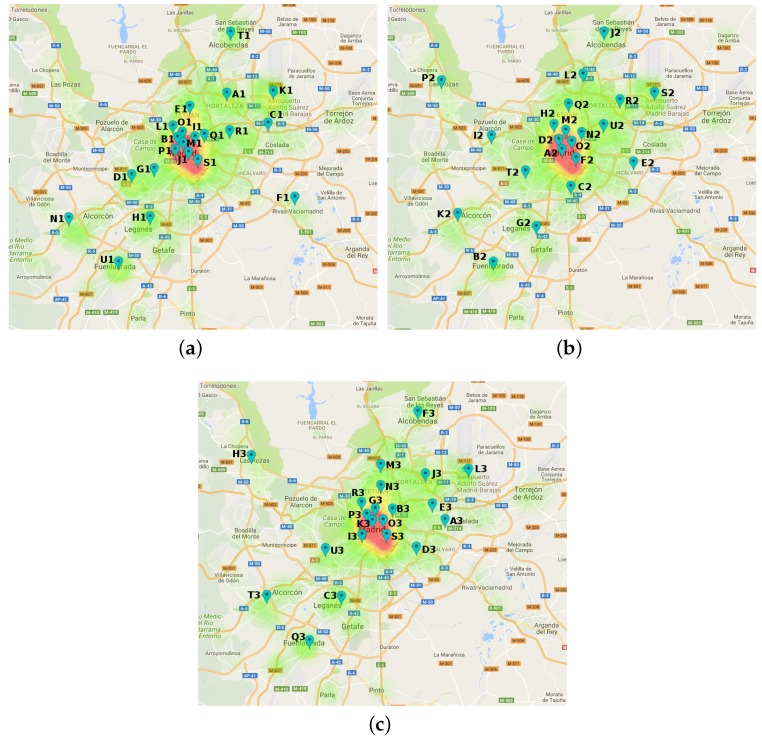
Centroids for MD dataset depicted as pins. The labelled ones are further discussed later. (**a**) night slot (1); (**b**) morning slot (2); (**c**) afternoon slot (3).

**Figure 8:**
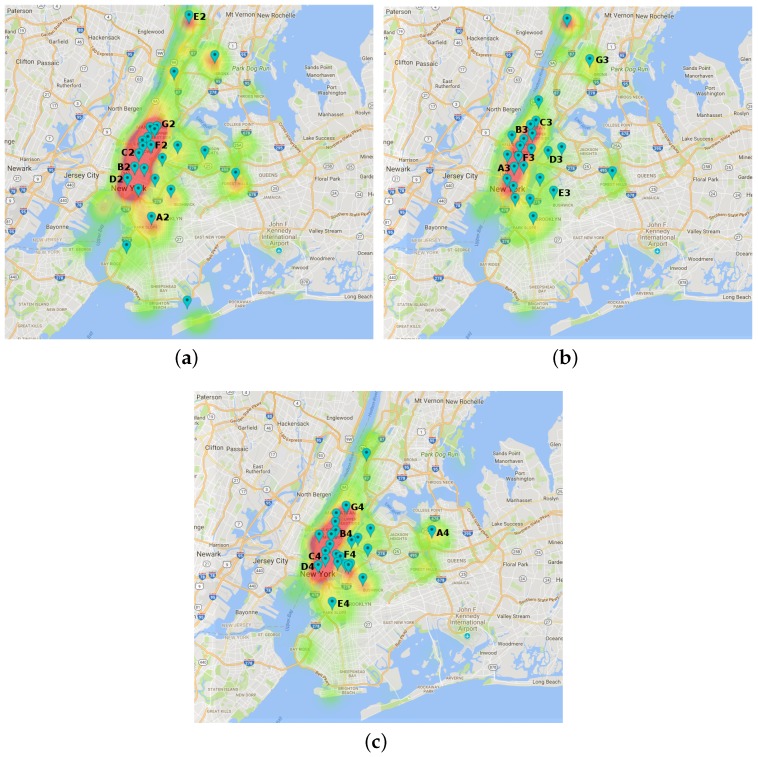
Centroids for NY dataset depicted as pins. The labelled ones are further discussed later. (**a**) morning slot (2); (**b**) afternoon slot (3); (**c**) evening slot (4).

Regarding the spatial distribution of the clusters, Figure 7 and Figure 8 depict the clusters’ centroids as location pins and the density of documents for three consecutive time slots per city. From these figures, we have made some interesting findings.

Concerning the MD dataset, Figure 7 shows that the spatial distribution of clusters meaningfully varies in the central area of the city. During the night period, downtown clusters are quite close to each other (Figure 7a). However, during the morning and afternoon periods, clusters are more separated. A possible explanation of this phenomenon might be due to the fact that night-life in Madrid basically occurs in the city center. To confirm this theory, we delve into the topics labelling some the the clusters in the central area of the city. In that sense, Table 7 shows the words for the two more relevant topics for some clusters at different time slots.

From such a table, we can see that some of the topic labels in the three clusters of the night period (B1, J1, M1) may refer to leisure night activities like cook, pow-wow, theatre or club. However, we also noticed that other clusters at different times slots were labelled with confusing and not very descriptive topics (like clusters F2 or B3). Since the MD dataset comprises documents from Twitter and Flickr platforms, we studied if there was any correlation between such platforms and the descriptive capabilities of the topics. For that reason, Table 8 shows the distribution of users with respect their OSN platform per cluster.

**Figure 9:**
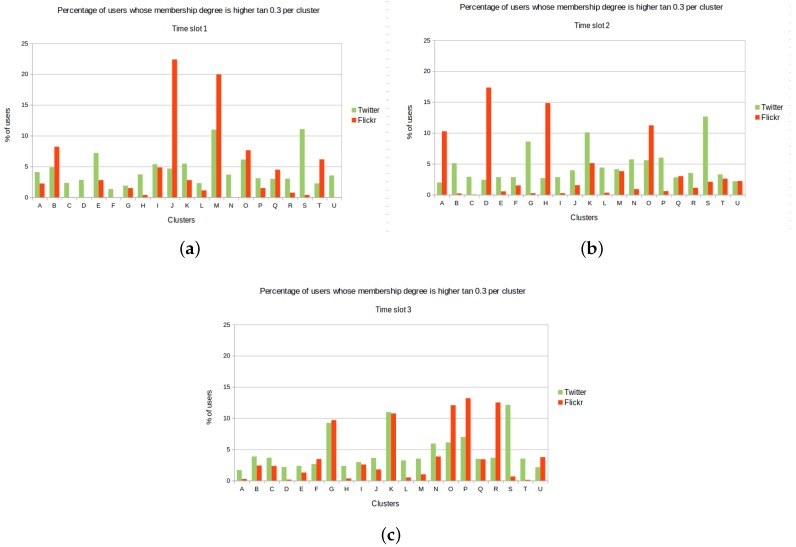
Distribution of OSN users per cluster in MD dataset. (**a**) night slot (1); (**b**) morning slot (2); (**c**) afternoon slot (3).

As we can see, the two clusters with the most descriptive topics in Table 7 (J1, M1) are the ones with the highest rate of Flickr users (see Figure 7a). On the contrary, the most poorly labelled clusters (F2 or B3) share two characteristics according to Figure 9a,b: (1) a reduced rate of users and (2) a higher percentage of Twitter users than Flickr ones. Consequently, according to these results, Flickr seemed a more accurate source for semantic information of clusters than Twitter.

As for the NY dataset, Figure 8 shows that the spatial distribution of clusters in NY is quite similar to MD as the highest density of clusters are concentrated in downtown city, in this case Manhattan, and a few of them are spread in the outskirts of the urban area.

In this case, we can see that a clusters D2 in the morning (Figure 8a), A3 in the afternoon (Figure 8b) and D4 (Figure 8c) in the evening slot are spatially located at a very similar coordinates. Consequently, in order to test the suitability of the topic assignment to clusters, Table 8 shows the two most relevant topics to each of the aforementioned clusters.

From this table, we can see that that the topic labelling meaningfully varies depending on the time slot. In more detail, we can see that certain topics’ words provide relevant information of certain activities in the area at different hours (e.g., run, athlete, tour) or meaningful landmarks (museum, metropolitan, art).

### 5.4. Pattern Detection

**Figure 10:**
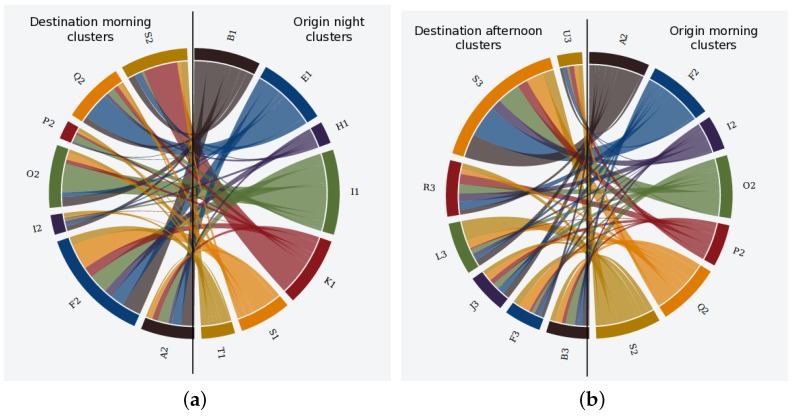
Mobility patterns for MD dataset. (**a**) transition from night to morning clusters; (**b**) transition from morning to afternoon clusters.

**Figure 11:**
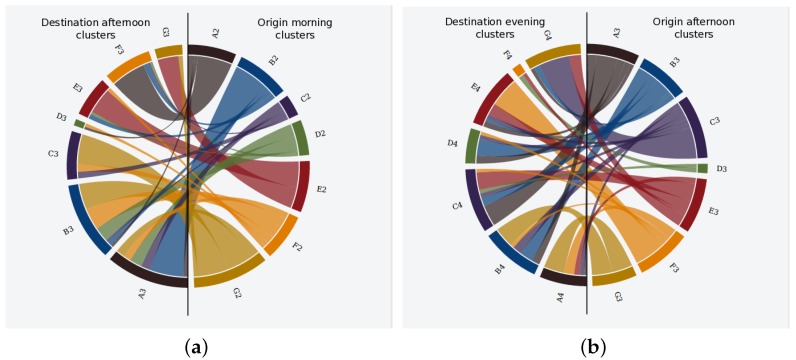
Mobility patterns for NY dataset. (**a**) transition from morning to afternoon clusters; (**b**) transition from afternoon to evening clusters.

Given the clusters generated in the previous section, the pattern discovery mechanism was executed so as to discover the transitions of users in between clusters. In that sense, Figure 10 and Figure 11 show the uncovered transitions for some of the clusters from the MD and NY datasets. In that sense, the origin clusters are located on the right side of the figures and the destination ones on the left side, so the flows moves from right to left.

If we observe both figures, we see that the transitions from the morning to the afternoon slot are much more messy in MD (Figure 10b) than in NY (Figure 11a). Concretely, the mobility patterns in NY in between such time slots can be roughly summarized as two trends: (1) a long northbound movement of people from the cluster B2 (located around Brooklyn borough according to the location of such cluster in Figure 8a) to cluster A3 in the center of the Manhattan area (see Figure 8b); (2) a short southbound displacement from cluster G2 to clusters B3 and C3.

Unlike such general trends, the mobility patterns between the morning and the afternoon periods in MD indicate that the city faces the movement of people at many different directions without any clear trends. However, the mobility patterns from night to morning clusters do show some remarkable trends for MD according to Figure 10a.

### 5.5. Predictor Performance

Finally, we have evaluated the performance of the predictor service by using both the MD and NY datasets. In that sense, we have split such datasets in a training and an evaluation dataset. With the former, we re-generated the mobility patterns again. Next, such patterns were used by the predictor that was fed with the evaluation set. In that sense, the proportion of the training and evaluation sets was set to 70%/30% of the original datasets.

#### 5.5.1. Measurements

For the measurement of the predictor, we have used two measurements: the detection rate (DR) and the prediction error (PE). DR counts the number of documents in the evaluation set for which at least one cluster is provided as prediction. By means of this factor, we intend to measure the coverage of the proposal. Therefore, it can be defined by means of the following formula:

$$\mathrm{DR}=\frac{|d_{tp}withprediction|}{|d_{tp}|}.$$

PE is the distance deviation for each prediction of a document. This measure indicates how far the system deviates from the actual next location of a user. For this case, we have made use of the haversine distance between the centroid’s location of the predicted cluster, $c_{dest}\left(l\right)$, and the actual location of the next document, $d_{next}\left(l\right)$. Hence, it can be defined by means of the following formula:

$$\mathrm{PE}=\mathrm{dist}(c_{dest}\left(l\right),d_{next}\left(l\right)).$$

#### 5.5.2. Results Discussion

Regarding the DR, Table 9 and Table 10 shows such parameters for both datasets split in time slots. For instance, Table 9 shows that the system was able to, given a document in the night slot, to predict the location of the user in the morning slot 63% of the time, or in the afternoon slot 70% of the time. The last column was Total DR. This way, the service was able to provide at least one prediction (in any destination time slot) to 87% of the documents in the morning slot at MD.

As we can see, for the two datasets, the highest DRs occur for when the afternoon and the evening are the destination slots. This is because such time slots comprise most of the documents for the MD dataset (see Table 5) that facilitates the prediction outcome.

In general terms, we can see that the system achieves a quite high DR, especially in NY. This is mainly because the fuzzy approach followed in the present work. In that sense, such approaches make it more easy for an incoming document to be assigned to an origin cluster and then infer the next movement of the user.

**Figure 12:**
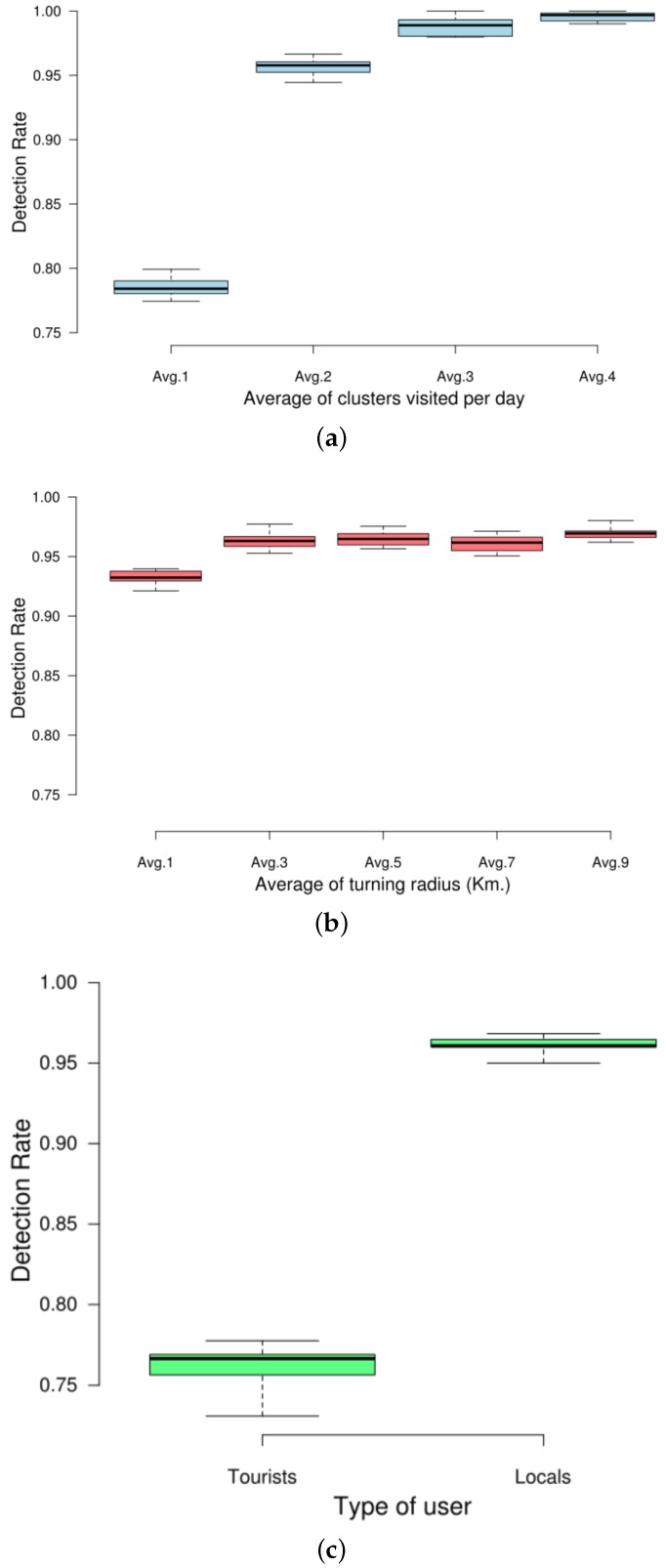
Detection rate of the predictor considering different factors related to users. (**a**) DR considering the avg. number of visited clusters per user per day; (**b**) DR considering the avg. radius of gyration of the target user’s trajectories; (**c**) DR considering tourist and resident users in NY and MD.

Moreover, we have also analysed the effect on our predictor of three different factors related to the target users. In particular, we focused on (i) the average number of clusters visited per day by a user, (ii) the average radius of gyration of a user’s trajectories and (iii) the type of user in terms of tourist or local resident. For this last distinction, we followed a time-based approach commonly accepted in the literature [43]. Basically, it divides the study period into 30-day blocks. If the users posted all their documents within a period of 30 days, the algorithm labelled them as visitors, but if they publish documents at intervals of more than 30 days, then it categorized them as residents. Figure 12 shows the DR considering the three aforementioned factors.

As far as the number of clusters is concerned (Figure 12a), we can see that there exists a direct correlation between the average number of clusters visited by a user and its predictability. In that sense, our approach is able to achieve a quite high DR when users tend to publish only two or more documents per day.

Regarding the radius of gyration (Figure 12b), we also appreciate a direct correlation between the size of the radius and the DR of the predictor. This is probably because users with trajectories having a large radius of gyration then publish more documents, and thus their trajectories are more distinguishable.

Concerning the dichotomy resident-tourist (Figure 12c), we can see the the DR is higher for locals than for tourists. The reason of this difference has to do with the time period each type of user spends in the city. Since tourists stay less days in the target city, the predictor has less information in order to compose a prediction, as it has more difficulties finding documents to compose patterns covering all the timeslots. This makes the DR of the predictor decrease.

**Figure 13:**
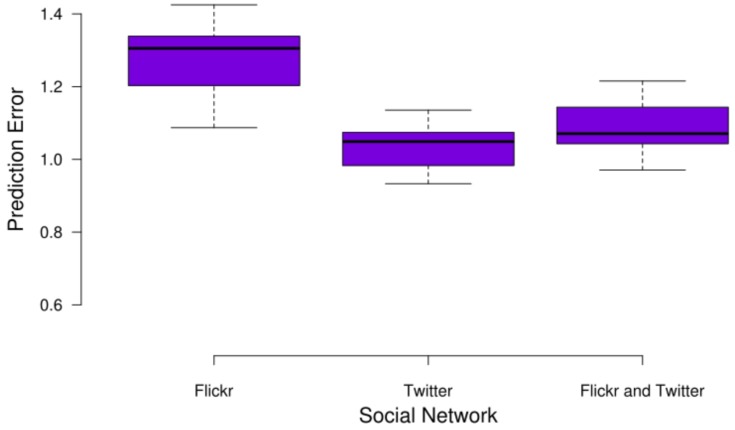
Average prediction error with respect to the target platform.

As for the PE, Figure 13 decomposes such measurement depending on the OSN platform to which the incoming document belongs. As we can see, the smaller errors are obtained for the Twitter documents, whereas the larger ones are obtained for the Flickr documents. This difference is due to the fact that Flickr documents usually belong to larger clusters in terms of space, which makes them farther from the centroid’s location.

**Figure 14:**
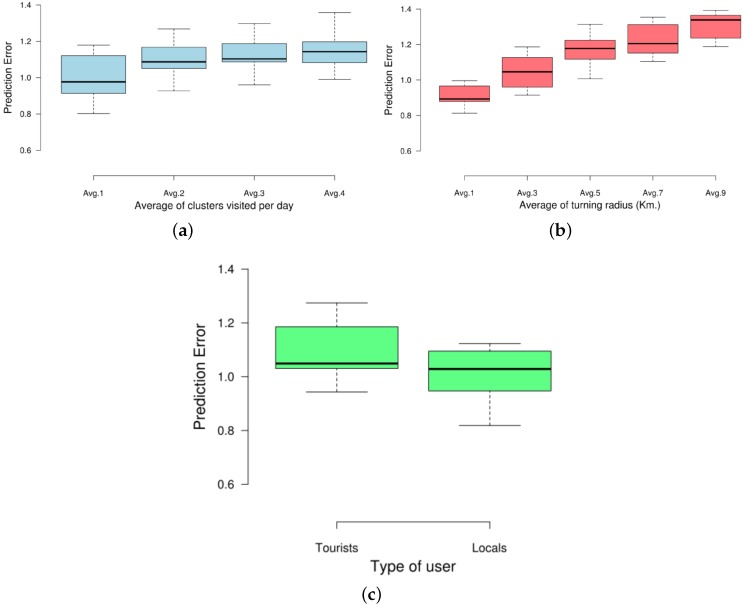
Prediction error of the predictor considering different factors related to users. (**a**) PE considering the avg. number of visited clusters per user per day; (**b**) PE considering the avg. radius of gyration of the target user’s trajectories; (**c**) PE considering tourist and resident users in NY and MD.

For this measurement, we have also studied the effect of the three aforementioned users’ factors. In that sense, the number of clusters does not meaningfully affect the DR of our approach (see Figure 14a). However, the radius of gyration negatively affects the predictor when taking large values (Figure 14b). Finally, our fuzzy-rule approach provided more accurate predictions for the residents in both cities than for tourists (Figure 14c). This is because the movement of locals tends to be more repetitive and focuses in quite close clusters in spatial terms. On the contrary, tourists usually follow more random paths across more spread clusters representing the different landmarks of the city.

## 6. Related Work

The study of human mobility on a large scale started in the 1950s with the creation of household travel surveys based on face-to-face, telephone or mail interaction. The limitations in terms of coverage of these surveys were overcome with the emergence of the Information and Communication Technologies (ICTs) [2]. This way, the usage of wireless sensors or traffic cameras giving insight into urban user dynamics defined a second era of large-scale mobility studies [44]. More recently, mobile phone networks have been adopted as another meaningful data source to come up with human mobility modelling [45]. In this context, OSNs have been studied for the last few years as a suitable source to extract mobility-related knowledge. From a utility perspective, the core of OSN-based works in the mobility mining discipline can be divided into three different lines of work (see Table 11).

Firstly, several works use OSNs as real-time data streams to detect certain events or incidents with respect the traffic of a city [46,60,61,62]. For this task, different classification algorithms, like Support Vector Machines or Random Forest, along with Natural Language Processing (NLP) techniques are combined. In brief, these works focus on detecting meaningful changes in the frequency and content of OSN documents submitted within a geographic area that might report a serious traffic situation.

A second line of work investigates the usage of heterogeneous OSN data to automatically detect regions within a city [47,48,49,63,64]. In this case, works explore OSN data as an enabler to discover how humans name places in order to assist attempts aimed at imitating this behaviour by computer systems. From the point of view of the applied data-mining techniques, these works can be generalized into two types: spatial clustering approaches that determines regions based on the intensity of human activity [14,47,48,49] and network-based approaches [64], where areas are determined with the intensity of human relations between regions.

Finally, a third line of work makes use of OSN data to compose mobility patterns that define the human movement in a geographic area. In this scope, several works follow a model-based approach able to classify or assign geo-tagged OSN documents to a particular mobility category or pattern [24,52,54]. In that sense, Latent Dirichlet Allocation (LDA) [24], Bayesian networks [52] or Origin–Destination (OD) matrices [54] are some examples of adopted models.

Our work can be enclosed in an alternative course of action for OSN-based mobility pattern discovery following a clustering-based approach. Basically, these works cluster the locations or paths followed by OSN users and then, on top of these clusters, make up the eventual mobility patterns [48,50,51,55]. In that sense, several clustering solutions have been proposed. In more detail, Ref. [48] makes use of the density-based clustering algorithm DBSCAN to firstly detect areas with high OSN activity using the spatial features of photos shared in Flickr. Then, a temporal clustering allows for uncovering the movement across these areas. Finally, the textual labels tagging the photos feed a semantic layer to make up a clouds of tags labelling each cluster. Similarly, Ref. [51] adapts the OPTICS algorithm, a density-based clustering for trajectories, to detect mobility patterns using the spatio-temporal features of documents from two different OSN platforms, Gowalla and Brightkite. Next, the Kullback–Leibler (KL) divergence is used as the similarity measurement to mine the evolution of these patterns through time. Ref. [50] envisions a non-negative matrix factorization to cluster profiling information of OSN users related to their activity score within the platform to capture the spatio-temporal features of their consecutive movements across a city.

Despite this variety, the usage of fuzzy clustering techniques with OSN data has not been fully exploited. For instance, Ref. [55] actually proposes a fuzzy modelling approach for human mobility mining. Nonetheless, several dissimilarities exist between that work and ours. Firstly, it uses the Fuzzy C-Means (FCM) clustering algorithm to uncover the OSN-active areas. In that sense, FCM is only able to generate spherical-shape clusters, whereas the GK algorithm, used in the present work, allows for generating clusters with different geometrical shapes. This is more convenient due to the heterogeneous distribution of OSN documents in urban areas. Secondly, whilst our approach fuses the spatio-temporal features and the textual content of OSN documents for the clusters’ identification, Ref. [55] only takes into account the spatial and temporal attributes of the documents. Finally, this work goes beyond the pattern discovery proposed in [55] by also developing a location predictor on top of the patterns. For the sake of completeness, we also mention the work in [59], which proposes a fuzzy version of a Multinominal Mixture Model (MMM) to detect the gender of the Twitter users on the basis of the textual content of their tweets.

Regarding location prediction, the anticipation of the future movement of a target individual is based on the idea that human mobility exhibits a high regularity, and, thus, predictability [6]. In this frame, our work also includes some innovative features with respect to existing literature related to OSN-based location predictors [16,56,57,58]. In this frame, most works make use of the spatio-temporal features of the documents in order to perform the prediction [56,57]. For example, like in [57], our approach also uses the spatio-temporal features of the documents of a user to make a prediction. However, the present work also takes under consideration the textual features of the documents in order to provide a prediction. In addition to that, whilst [57] proposes a real-time system orchestrated by means of event-based rules, our work combines two steps: (i) an offline one to generate the clusters and the underlying mobility patterns and (ii) an online step where the patterns are used to generate a prediction in real time. This way, we avoid the convergence period problem that the mechanism in [57] suffers from. Another difference exists regarding the particular prediction algorithm, while the work in [57] makes use of a Fallback Markov Model, we rely on fuzzy rules. In that sense, these rules provide more flexible capabilities to deal with uncertainty than the aforementioned Markov model.

A different approach is put forward in [16] that considers the spatial distribution of words of OSN documents to predict the next location of an OSN users. Like our proposal, Ref. [58] considers the three dimensions of an OSN document: temporal, location and textual features. By following a Bayesian-network approach, the proposed system is able to forecast the next location and activity of a user by also taking into account temporal factors. However, in our case, we follow a fuzzy-rule system, which is able to deal with the uncertainty inherent to OSN data.

## 7. Conclusions

The study of human dynamics is paramount for the development of innovative services in the context of large cities. In that sense, OSN platforms have arisen as a cost-effective data source to extract human-generated mobility data. For that reason, the research community has provided several solutions to mine mobility patterns by using such OSN data. However, the proper management of their inherent uncertainty and the full analysis of all their characteristics is still an open issue.

For that reason, the present work puts forward an innovative fuzzy model for human dynamics that solely relies on OSN data. By means of well-established fuzzy algorithms and classifiers, we have developed a mechanism able to extract the social areas of a city and the mobility flows among them. Furthermore, we made use of the textual content of OSN documents in order to semantically enrich the discovered areas. On top of such a solution, a prediction service has been implemented in order to anticipate future movements of city dwellers. The evaluation study has shown the feasibility of the proposal by detecting the mobility patterns in two different cities and the convenience of the semantic enrichment of the clusters.

Finally, future work will focus on including metrics to asses the accuracy of the semantic labelling of the clusters. In that sense, the usage of well-known ontologies like Wordnet is foreseen. Moreover, other repositories reporting land-use data will be studied in order to allow the static and dynamic labelling of the uncovered clusters. 

## Figures and Tables

**Table 1:** Framework notation.

Acronym	Meaning
A	Target urban area to detect its mobility patterns
*d*	OSN document
$d_{f}$	Filtered OSN document
$D_{f}$	Set of filtered OSN documents
$D_{f}\left(x\right)$	Sub-set of filtered OSN documents only including attribute *x*
TP	Set of latent topics of $D_{f}$
$d_{tp}$	Filtered OSN document with latent topics
$d_{tp}\left(x\right)$	Attribute *x* of a filtered document with latent topics
$D_{tp}$	Set of filtered OSN documents with latent topics
$D_{tp}\left(x\right)$	Sub-set of filtered OSN documents with latent topics only including attribute(s) *x*
$D_{tp}^{x}$	Sub-set of filtered OSN documents with latent topics only including documents from user *x*

**Table 2:** Examples of individual mobility patterns.

User Pattern	Time Slot				
	1	2	3	4	5
$p_{user1}$	$A_{1}$	$B_{2}$	$C_{3}$	$S_{4}$	$D_{5}$
$p_{user2}$	$A_{1}$	$A_{2}$	$V_{3}$	$L_{4}$	$Y_{5}$
$p_{user3}$	∅	$B_{2}$	$Z_{3}$	$S_{4}$	$Q_{5}$
$p_{user4}$	$D_{1}$	$E_{2}$	$C_{3}$	$N_{4}$	$R_{5}$
$p_{user5}$	$A_{1}$	$B_{2}$	$C_{3}$	$S_{4}$	$F_{5}$

**Table 3:** Evaluation datasets.

Feature	Madrid (MD)	New York (NY)
OSN Platforms	Twitter + Flickr	Flickr
Time period	22 January 2016 to 21 April 2016	22 January 2011 to 21 April 2011
Covered area (km${}^{2}$)	605	1214
Geo-tagged documents/users	280,860/43,532	38,797/1474

**Table 4:** Datasets cleaning results. Number in brackets are the percentage with respect to the raw documents.

Feature	Madrid (MD)	New York (NY)
Spam documents/users	89,292 (32%)/30	3927 (10%)/13
Final documents/users	191,568 (68%)/43,502	34,870 (90%)/1461

**Table 5:** Distribution of documents per time slot. Number in brackets are the percentage with respect to the total.

Timeslot	MD	NY
Night (1)	12,527 (8%)	4415 (13%)
Morning (2)	37,061 (19%)	8233 (24%)
Afternoon (3)	49,847 (26%)	9523 (27%)
Evening (4)	60,850 (31%)	10,252 (29%)
Late evening (5)	31,283 (16%)	2447 (7%)
Total	191,568	34,870

**Table 6:** Number of automatically detected fuzzy clusters.

Dataset	Number of Clusters
MD	20
NY	23

**Table 7:** Cluster topic samples from the MD dataset.

Cluster	Topic	Words
Night period		
B1	1	birthday, cook, street
	2	go, pow-wow, best
J1	1	theatre, wonderful, start
	2	night, sound, rest
M1	1	crazy, club, joy
	2	gift, door, sun
Morning period		
A2	1	good, morning, smile
	2	airport, go, bye
D2	1	park, sun, door
	2	day, years, last
F2	1	Spain, beautiful, best
	2	happy, sunday, go
Afternoon period		
S3	1	do, paint, form
	2	house, day, rain
B3	1	back, camp, record
	2	palace, crystal, common

**Table 8:** Cluster topic samples from the NY dataset.

Cluster	Topic	Words
Morning period		
D2	1	photo, subway, peple
	2	day, patrick, st.
Afternoon period		
A3	1	run, athlete , ny
	2	museum, metropolitan, art
Evening period		
D4	1	janet, tour, jackson
	2	central, park, white

**Table 9:** Detection rate of the predictor per time slot for MD dataset. The best results per origin time slot are marked in bold.

From/To	Night (1)	Morning (2)	Afternoon (3)	Evening (4)	Late Evening (5)	Total
Night (1)	-	0.63	0.70	**0.73**	0.64	0.85
Morning (2)	0.40	-	0.75	**0.76**	0.57	0.87
Afternoon (3)	0.36	0.61	-	**0.73**	0.56	0.84
Evening (4)	0.37	0.60	**0.70**	-	0.59	0.83
Late evening (5)	0.39	0.54	0.66	**0.71**	-	0.82
Average						0.84

**Table 10:** Detection rate of the predictor per time slot for NY dataset. The best results per origin time slot are marked in bold.

From/To	Night (1)	Morning (2)	Afternoon (3)	Evening (4)	Late Evening (5)	Total
Night (1)	-	0.54	0.63	**0.77**	0.63	0.92
Morning (2)	0.53	-	**0.87**	0.76	0.30	0.93
Afternoon (3)	0.54	**0.82**	-	0.78	0.39	0.95
Evening (4)	0.57	0.58	**0.83**	-	0.61	0.92
Late evening (5)	0.80	0.38	0.74	**0.94**	-	0.98
Average						0.94

**Table 11:** Main features of other proposals.

Ref.	OSN Platform	Data	Method/Algorithm	Outcome
[46]	Flickr	temporal, location, textual	Single-pass incremental clustering	event detection
[47]	Gowalla	location	DBSCAN, Grivan–Newman	land use
[48]	Flickr	temporal, location, textual	DBSCAN	land use
[49]	Foursquare	temporal, location	Spectral clustering	landuse
[14]	Twitter	location	Self-organizing map, K-means	land use
[24]	Twitter	location	EM-algorithm, LDA	mobility patterns
[25]	Twitter	temporal, location	DBSCAN, temporal clustering	mobility patterns
[50]	Foursquare	temporal, location, friendship	Non-negative matrix factorization	mobility patterns
[51]	Gowalla, Brightkite	temporal, location	OPTICS, KL divergence	mobility patterns
[52]	Sine Weibo	textual, friendship	Naïve Bayes	mobility patterns
[53]	Twitter	temporal, location, profile	OD matrix analysis	mobility patterns
[54]	Twitter	temporal, location	Gravity model	mobility patterns
[55]	Twitter	temporal, location	FCM	mobility patterns
[16]	Twitter	textual	Naïve Bayes, SVN classifier	prediction
[56]	Flickr	temporal, location	ad hoc Gravity model	prediction
[57]	Twitter	temporal, location	ad hoc DBSCAN, Markov model	prediction
[58]	Twitter	temporal, location, textual	Bayesian network	prediction
[59]	Twitter	textual	Fuzzy MMM	gender identification
Our Proposal	Twitter, Flickr	temporal, location, textual	GK, AHC	mobility pattern, prediction

## Appendix A. Fuzzy within-Cluster (*S_W_*) and between-Cluster (*S_B_*) Scatter Matrices

$$S_{W}=\sum_{i=1}^{c}\sum_{k=1}^{n}{\left(u_{ik}\right)}^{m}\left(x_{k}-v_{i}\right){\left(x_{k}-v_{i}\right)}^{T},$$

$$S_{B}=\sum_{i=1}^{c}\left(\sum_{k=1}^{n}{\left(u_{ik}\right)}^{m}\right)\left(v_{i}-\bar{v}\right){\left(v_{i}-\bar{v}\right)}^{T},$$

where $v_{i}$ are the clusters centroids calculated by

$$v_{i}=\frac{\sum_{k=1}^{n}u_{ik}^{m}x_{k}}{\sum_{k=1}^{n}u_{ik}^{m}},$$

and the vector $\bar{v}$ is the instances mean taking into account their membership in each cluster:

$$\bar{v}=\frac{\sum_{i=1}^{c}\sum_{k=1}^{n}{\left(u_{ik}\right)}^{m}x_{k}}{\sum_{i=1}^{c}\sum_{k=1}^{n}{\left(u_{ik}\right)}^{m}}.$$

## Appendix B. Cluster Validity Criterium

The $S_{W}$ and $S_{B}$ matrices must be taken into account to choose a suitable number of centroids *c* to configure GK. Firstly, *c* must minimize the trace of $S_{W}$ to get compact clusters. Secondly, *c* must maximize the trace of $S_{B}$ to get a desirable separation among the clusters. In other words, it must minimize the trace of $S_{W}-S_{B}$.

Regarding the setting of the weighting exponent *m*, we have to take into account its range of possible values [1,∞). The larger *m* is, the fuzzier the clusters associated to the centroids are. Hence, the suitable value for *m* has to be far enough from its two limits. For the purpose of getting this value, the fuzzy total scatter matrix ($S_{T}$) is defined as the addition of $S_{W}$ and $S_{B}$:

$$S_{T}=S_{W}+S_{B}.$$

According to [65], the trace of $S_{T}$ decreases monotonically from a constant value *K* to zero, as *m* varies from one to infinite. The value of *K* is determined only by the set of examples [31]:

$$K=trace\left(\sum_{k=1}^{l}\left[\left(d_{tp}^{k}-\frac{1}{n}\sum_{k=1}^{n}d_{tp}^{k}\right){\left(d_{tp}^{k}-\frac{1}{n}\sum_{k=1}^{n}d_{tp}^{k}\right)}^{T}\right]\right).$$

Consequently, a value of *m* is suitable if it keeps the trace of $S_{T}$ somewhere in the middle of the domain [0,K]. The trace of $S_{T}$ is a function of the number of centroids *c* and the weighting exponent *m*, so an iterative process can be defined to get both *c* and *m*:

1. Calculate *K* for the given set of OSN documents.
2. Select a value of *m* by some criteria or heuristic.
3. Calculate the trace of $S_{W}-S_{B}$ for different values of *c*. Choose the *c* with the best trace.
4. Calculate the trace of $S_{T}$ with the chose values of *m* and *c*. If this value is far enough from the limits [0,K], then finish. Otherwise, go to 2.

If the instances of the data set are very near each other, and *m* is near 2, then there will be a high degree of overlapping. In our setting, *m* is initialized with the value 1.0; then, it is increased by 0.05 in every iteration. In this way, we first check low values of *m*, so that we get clusters with little overlapping.

Finally, this iterative process will be executed for each of the five instances of GK. Hence, the set OSN documents to calculate *K* in the first step only includes the ones with $\delta_{j}\geq 0.5$ for the target time slot of the GK instance.
